# COMPARATIVE TRANSCRIPTOMICS AND PROTEOMICS ACROSS SPECIES

**DOI:** 10.1093/geroni/igad104.1424

**Published:** 2023-12-21

**Authors:** Vera Gorbunova

**Affiliations:** University of Rochester, Rochester, New York, United States

## Abstract

Mammals differ more than 100-fold in maximum lifespan. Here, we conducted comparative transcriptomics on 26 species with diverse lifespans. We identified thousands of genes with expression levels negatively or positively correlated with a species’ maximum lifespan (Neg- or Pos-MLS genes). Neg-MLS genes are pri- marily involved in energy metabolism and inflammation. Pos-MLS genes show enrichment in DNA repair, microtubule organization, and RNA transport. Expression of Neg- and Pos-MLS genes is modulated by interventions, including mTOR and PI3K inhibition. Regulatory networks analysis showed that Neg-MLS genes are under circadian regulation possibly to avoid persistent high expression, whereas Pos-MLS genes are tar- gets of master pluripotency regulators OCT4 and NANOG and are upregulated during somatic cell reprogramming. Pos-MLS genes are highly expressed during embryogenesis but significantly downregulated after birth. This work provides targets for anti-aging interventions by defining pathways correlating with longevity across mammals and uncovering circadian and pluripotency networks as central regulators of longevity. I will also discuss integrating this transcriptome and proteome data.

